# Relieving the pressure: the effect of active carbon dioxide aspiration on postoperative pain after laparoscopic cholecystectomy

**DOI:** 10.1186/s12893-026-03567-y

**Published:** 2026-02-04

**Authors:** Osman Gökhan Gökdere, Bahadır Öndeş, Ahmet Aydın

**Affiliations:** 1 Department of General Surgery, Faculty of Medicine, Malatya Turgut Özal University, Malatya, Türkiye; 2 Department of Anesthesiology and Reanimation, Faculty of Medicine, Malatya Turgut Özal University, Malatya, Türkiye

**Keywords:** Laparoscopic cholecystectomy, Active gas aspiration, Carbon dioxide evacuation, Postoperative pain, Visual analog scale

## Abstract

**Background:**

Residual carbon dioxide (CO₂) after laparoscopic cholecystectomy (LC) may contribute to early postoperative pain. Active aspiration of CO₂ has been proposed as a simple adjunct, but large cohort data remain limited.

**Methods:**

This non-randomized prospective cohort study included 270 patients undergoing LC between May 2025 and October 2025. Patients were allocated to either the active aspiration group (*n* = 135) or the passive evacuation group (*n* = 135). Pain was evaluated using the Visual Analog Scale (VAS) at 4 and 24 h. Statistical analyses included chi-square tests, independent and paired t-tests, and Pearson correlation. A priori power analysis determined that 266 patients (133 per group) were required (effect size d = 0.40, α = 0.05, power = 0.90).

**Results:**

The aspiration group had significantly lower pain scores at both 4 h (3.48 ± 1.08 vs. 5.30 ± 1.60; *p* < 0.001) and 24 h (1.69 ± 0.94 vs. 3.59 ± 1.38; *p* < 0.001). Active aspiration was associated with lower pain scores at both time points (*p* < 0.001).

**Conclusion:**

Active evacuation of residual CO₂ was associated with lower postoperative pain scores following LC. Given the non-randomized design, causal inference is limited, and the findings should be interpreted cautiously.

**Supplementary Information:**

The online version contains supplementary material available at 10.1186/s12893-026-03567-y.

## Introduction

 Laparoscopic cholecystectomy (LC) is widely recognized as the gold-standard surgical technique for the treatment of benign gallbladder diseases, offering several advantages over open surgery, including reduced postoperative pain, shorter hospital stay, faster recovery, and improved cosmetic outcomes [1]. Despite these well-established benefits, a considerable proportion of patients continue to experience moderate-to-severe pain during the first 24 h after surgery [2]. This postoperative discomfort may delay ambulation and oral intake, prolong hospital stay, and negatively affect overall patient satisfaction [3].

Post-laparoscopic pain is multifactorial in origin and includes parietal pain from trocar incisions, visceral pain related to peritoneal stretching, and referred shoulder pain associated with diaphragmatic irritation [4]. Additional contributing factors—such as inflammatory activation, intra-abdominal acidosis, and systemic absorption of carbon dioxide (CO₂)—may further intensify peritoneal irritation and visceral discomfort. Among these components, shoulder pain is often reported as particularly distressing. The principal mechanism involves residual CO₂ retained within the peritoneal cavity after pneumoperitoneum, which may irritate the diaphragm and stimulate the phrenic nerve, resulting in referred shoulder pain [5]. This mechanism has been described in both gynecologic and general laparoscopic procedures, with pain severity reported to correlate with the volume of retained CO₂ [6].

During laparoscopic procedures, CO₂ insufflation is required to achieve adequate abdominal distension and optimal visualization of the operative field. However, incomplete evacuation of CO₂ at the end of surgery may contribute to diaphragmatic tension and peritoneal irritation, thereby prolonging postoperative discomfort. Several intraoperative strategies have been proposed to mitigate this effect, including low-pressure pneumoperitoneum, pulmonary recruitment maneuvers, patient positioning adjustments, and intraperitoneal irrigation. While some of these approaches demonstrate partial effectiveness, many require additional time, resources, or specialized equipment, which may limit their routine application [7].

Active aspiration of residual CO₂ before trocar removal has therefore gained attention as a simple and potentially cost-effective technique to facilitate gas evacuation. By mechanically removing retained CO₂ under direct visualization, this approach may reduce diaphragmatic irritation and postoperative discomfort [8]. Although prior randomized and observational studies suggest that active aspiration may be associated with lower postoperative pain intensity, findings across studies remain heterogeneous, often due to small sample sizes, methodological differences, and variations in perioperative practice [9]. Consequently, large-scale cohort studies evaluating the real-world performance of active CO₂ aspiration remain limited.

The present study aimed to evaluate the association between active gas aspiration performed at the end of LC and postoperative pain intensity at the 4th and 24th postoperative hours in a large patient cohort. In addition, relationships between pain scores and demographic variables such as age and sex were explored. Given the non-randomized prospective cohort design, the study was intended to assess associations rather than establish causal effects. We hypothesized that active gas aspiration would be associated with lower early and late postoperative pain scores.

## Materials and methods

### Study design and ethical approval

This prospective, non-randomized cohort study was conducted at the Department of General Surgery, Malatya Turgut Özal University Faculty of Medicine, between May 2025 and October 2025. The study was designed to evaluate associations between active CO₂ aspiration and postoperative pain under routine clinical practice conditions. The study adhered to the ethical principles of the Declaration of Helsinki and received approval from the Institutional Clinical Research Ethics Committee of Malatya Turgut Özal University (Approval No: E-30785963-020-286331; Date: February 5, 2025). Written informed consent was obtained from all participants prior to enrollment.

### Patient selection

Patients aged 18–91 years undergoing elective LC were screened for eligibility. Inclusion criteria were a diagnosis of symptomatic cholelithiasis or chronic cholecystitis, American Society of Anesthesiologists (ASA) physical status classification I–II, and normal preoperative liver function test results.

Exclusion criteria included acute cholecystitis, choledocholithiasis, Mirizzi syndrome, prior upper abdominal surgery, intraoperative complications related to pneumoperitoneum, or the need for additional surgical procedures during the postoperative period. Patients with acute cholecystitis and cases complicated by intraoperative gallbladder perforation or bile spillage were excluded to minimize baseline heterogeneity in postoperative pain outcomes. Acute inflammatory conditions and chemical peritoneal irritation may independently increase postoperative pain through mechanisms unrelated to residual CO₂ volume, thereby confounding the assessment of pneumoperitoneum-related pain. Intraoperative gallbladder perforation and bile spillage were not systematically recorded in a standardized manner; therefore, these events could not be analyzed as covariates and were handled by protocol-based exclusion to reduce heterogeneity.

A total of 270 patients met the eligibility criteria and were enrolled. Individual patient randomization was not performed. Instead, group allocation was determined by a predefined weekly institutional practice in which active CO₂ aspiration and passive evacuation were alternated on a weekly basis. Thus, allocation was based on operative week rather than surgeon preference or intraoperative judgment. All procedures were performed by the same two senior surgeons to ensure technical consistency. Patients receiving active gas aspiration constituted the aspiration group (*n* = 135), while those undergoing passive evacuation alone formed the control group (*n* = 135). The non-randomized allocation strategy is acknowledged as a potential source of selection bias.

### Surgical technique

All procedures were performed under general anesthesia using a standard four-trocar LC technique by the same experienced surgical team. Patients were positioned in the reverse Trendelenburg position with slight left tilt to optimize exposure of Calot’s triangle. Pneumoperitoneum was established using CO₂ at a pressure of 12 mmHg and a flow rate of 5 L/min. Identical insufflation and desufflation protocols were applied to all patients.

As part of institutional routine practice, a 5-mm Jackson–Pratt (JP) drain was placed in the subhepatic space after inspection of the surgical field and before final desufflation. To prevent unintended evacuation of pneumoperitoneum gas, the drain remained clamped and non-functional until all trocars had been removed. The Jackson–Pratt drain (JP drain) is a closed-suction system positioned in the subhepatic compartment, anatomically distinct from the hepato-diaphragmatic recess where residual CO₂ typically accumulates. Therefore, drain placement was not expected to influence residual intraperitoneal gas volume in either group. The drain was used solely for postoperative monitoring of bile leakage or hemorrhage.

In the active aspiration group, CO₂ insufflation was discontinued and the patient returned to the supine position. Under direct laparoscopic visualization, residual CO₂ was actively aspirated through a 5-mm trocar positioned over the right hepatic lobe using a standard suction device. Aspiration was continued until the omentum visibly contacted the anterior abdominal wall, indicating near-complete evacuation of free intraperitoneal gas.

In the control group, no active suction was applied. Gas evacuation occurred passively during standardized instrument and trocar withdrawal. No additional maneuvers, such as diaphragmatic compression, pulmonary recruitment, or positional modifications, were performed in either group.

### Pain assessment

Postoperative pain was assessed using a 10-point Visual Analog Scale (VAS), where 0 indicated no pain and 10 indicated the worst imaginable pain. Pain scores were recorded at rest and during mobilization at the 4th and 24th postoperative hours by ward nurses who were not involved in the surgical procedures and were blinded to group allocation. All patients received a standardized postoperative analgesia regimen consisting of intravenous dexketoprofen trometamol (50 mg every 12 h) according to the institutional protocol, and no opioids or additional analgesics were administered prior to VAS assessments.

Pain was evaluated as a global VAS score reflecting the overall postoperative pain experience. The primary outcome was defined as the arithmetic mean of rest and mobilization VAS scores, providing a composite measure of overall pain burden that captures both static and dynamic pain conditions and allows comparability across patients. Rest and mobilization scores were also analyzed separately in secondary analyses to assess consistency of findings. Pain was not subdivided into shoulder, incisional, or visceral components, as postoperative pain in routine clinical practice is commonly perceived and reported as a composite experience.

### Statistical analysis

Statistical analyses were performed using IBM SPSS Statistics version 26 (IBM Corp., Armonk, NY, USA). Continuous variables were summarized as mean ± standard deviation (SD) or median (minimum–maximum), while categorical variables were expressed as frequencies and percentages.

Normality was evaluated using skewness–kurtosis statistics and visual inspection of histograms and Q–Q plots. Between-group comparisons were conducted using chi-square tests for categorical variables and independent-samples t-tests for continuous variables. Paired-samples t-tests were used to assess within-group changes over time. Pearson correlation analysis was applied to explore associations between continuous variables.

Given the limited availability of consistently recorded clinical covariates, multivariable regression modeling was not performed. An exploratory path analysis examining associations between aspiration status, age, sex, and postoperative pain was conducted and is presented in the Supplementary Material. This analysis was exploratory in nature and not intended to support causal inference. A two-tailed p-value < 0.05 was considered statistically significant. Because several clinically relevant covariates were not consistently documented, any multivariable regression model would have been incomplete and potentially misleading; therefore, we did not perform adjusted modelling and report unadjusted group comparisons as the primary analysis.

### Power analysis

Sample size estimation was performed using GPower version 3.1.9 (Universität Düsseldorf, Germany). Assuming a medium effect size (Cohen’s d = 0.40), a statistical power of 0.90, and a two-sided α level of 0.05, a minimum of 133 patients per group (total *n* = 266) was required. The final sample size of 270 patients provided an achieved power of 0.90, confirming adequate power to detect between-group differences in postoperative pain scores.

## Results

### Descriptive characteristics

A total of 270 patients were included in the study, with 135 patients in each group.

Baseline demographic characteristics were comparable between groups with respect to age and sex (Table 1).

**Table 1:** Demographic characteristics of patients with and without active gas aspiration during laparoscopic cholecystectomy

Variable	Without Active Aspiration (*n* = 135)	With Active Aspiration (*n* = 135)	*p*-value
**Sex, n (%)**			0.125
Male	21 (15.6%)	32 (23.7%)	
Female	114 (84.4%)	103 (76.3%)	
Age category, n (%)			
< 50 years	68 (50.4%)	64 (47.4%)	0.715
≥ 50 years	67 (49.6%)	71 (52.6%)	
Age (years), mean ± SD (range)	49.13 ± 13.66 (19–77)	48.44 ± 14.55 (18–91)	0.689

The proportion of male patients was slightly higher in the active aspiration group (23.7%) compared with the control group (15.6%); however, this difference was not statistically significant (*p* = 0.125). Similarly, no statistically significant differences were observed between groups with respect to age distribution or mean age (*p* > 0.05 for all comparisons). These findings suggest that the two groups were broadly comparable in terms of baseline demographic characteristics, although differences in unmeasured clinical variables cannot be excluded due to the non-randomized study design.

### Postoperative pain scores

Comparisons of postoperative pain intensity at the 4th and 24th postoperative hours are summarized in Table 2. Unadjusted mean differences and corresponding 95% confidence intervals are presented.

**Table 2:** Comparison of postoperative pain intensity between groups

Time	Without Active Aspiration (Mean ± SD)	With Active Aspiration (Mean ± SD)	Mean Difference (95% CI)	*p*-value
4th postoperative hour	5.30 ± 1.60 (3–10)	3.48 ± 1.08 (1–5)	1.82 (1.45–2.19)	< 0.001
24th postoperative hour	3.59 ± 1.38 (1–8)	1.69 ± 0.94 (0–5)	1.90 (1.56–2.24)	< 0.001

Values are presented as mean ± standard deviation (SD). The 95% confidence intervals were calculated using independent-samples t-tests

Postoperative pain scores decreased significantly from the 4th to the 24th hour in both groups (*p* < 0.05). Patients in the active aspiration group reported lower VAS scores than those in the control group at both time points (*p* < 0.001 for both comparisons). These results indicate an observed association between active CO₂ aspiration and lower postoperative pain scores; however, causal inference cannot be made due to the observational nature of the study.

### Correlation analysis

Active gas aspiration demonstrated a moderate negative correlation with pain scores at both the 4th and 24th postoperative hours (*p* < 0.001). Sex showed a weak correlation with pain at the 4th hour, while age was not significantly correlated with pain scores at either time point. These correlations represent statistical associations and should be interpreted cautiously, as several clinically relevant determinants of postoperative pain were not available for analysis (Table 3).

**Table 3:** Correlation between postoperative pain levels and active gas aspiration, sex, and age

Time	Variable	*r*	*p*-value
4th hour	Active gas aspiration	-0.555**	< 0.001
	Sex	0.209**	0.001
	Age	0.029	0.635
24th hour	Active gas aspiration	-0.628**	< 0.001
	Sex	0.110	0.072
	Age	0.062	0.306

### Supplementary analysis

An exploratory path analysis evaluating associations between aspiration status, age, sex, and postoperative pain is presented in the Supplementary Material. This analysis was performed for exploratory purposes only and was not intended to support causal inference.

## Discussion

This prospective cohort study evaluated the association between active aspiration of residual CO₂ and postoperative pain following LC in a large patient population. Active CO₂ aspiration was associated with lower postoperative pain scores at both early (4th hour) and late (24th hour) postoperative time points. In contrast, demographic variables such as sex and age demonstrated only weak or no associations with postoperative pain intensity. These findings suggest that aspiration status showed a more consistent association with postoperative pain outcomes than basic demographic factors in this cohort; however, causality cannot be inferred.

The modest difference observed between male and female patients during the early postoperative period may reflect sex-related differences in pain perception or inflammatory response. Previous studies have suggested that hormonal factors, including estrogen and progesterone, may influence nociceptive thresholds and central pain processing. Nevertheless, given the non-randomized design of the present study and the absence of detailed hormonal or biochemical data, such interpretations remain speculative and should be considered exploratory.

Although CO₂ evacuation occurs in both groups, passive desufflation may leave residual gas trapped in the subphrenic and hepato-diaphragmatic recesses, particularly when evacuation relies solely on trocar removal. This concept is supported by randomized clinical trials demonstrating that active aspiration under direct visualization results in significantly lower early postoperative pain compared with passive desufflation alone. In a randomized study by Abdelsamad et al., active aspiration was associated with reduced postoperative pain scores despite identical surgical expertise and pneumoperitoneum protocols [8]. Similarly, a recent systematic review and meta-analysis of randomized controlled trials reported a consistent reduction in postoperative pain intensity with active gas aspiration techniques, supporting the notion that targeted evacuation improves the anatomical completeness of CO₂ removal beyond passive trocar venting [9].

These findings suggest that the benefit of active aspiration is not merely related to surgeon experience, but rather to the more effective clearance of residual CO₂ from dependent subphrenic spaces, which are less reliably emptied by passive desufflation alone.Active aspiration under direct visualization may facilitate more complete removal of localized residual CO₂ pockets that are not effectively eliminated by passive methods alone. Previous studies have demonstrated that even small volumes of retained CO₂ beneath the diaphragm can trigger phrenic nerve irritation and referred shoulder pain. Therefore, the observed association may reflect differences in the efficiency and anatomical completeness of CO₂ evacuation rather than the mere presence or absence of desufflation. In routine practice, trocar withdrawal may not reliably clear gas from dependent subphrenic recesses; direct suction may improve the anatomical completeness of evacuation even when procedures are performed by experienced laparoscopists. This difference is consistent with the concept that residual gas distribution is non-uniform and that targeted suction may reduce subphrenic CO₂ pockets that are less responsive to passive venting. Randomized trials comparing active aspiration with other evacuation maneuvers have reported lower early postoperative pain scores in the active aspiration arms. In addition, a recent systematic review and meta-analysis of randomized controlled trials reported an overall reduction in postoperative pain with active gas aspiration techniques. These findings support the biological plausibility that targeted evacuation may reduce subphrenic residual CO₂ pockets that are less effectively cleared by passive venting.

Shoulder pain represents a key mechanistic component of CO₂-related diaphragmatic irritation; however, postoperative pain is commonly experienced as a composite phenomenon. The use of a global VAS score was therefore chosen to reflect patient-centered pain perception, with the acknowledged limitation of reduced mechanistic discrimination.

### Comparison with previous literature

Postoperative pain following laparoscopic surgery is multifactorial and includes incisional, visceral, and referred (shoulder) pain components. Retained CO₂ beneath the diaphragm may contribute to peritoneal irritation and phrenic nerve stimulation, resulting in shoulder pain [10]. Passive evacuation of intraperitoneal gas may be insufficient, and several studies have therefore explored active aspiration or alternative decompression techniques to reduce residual CO₂ [11].

Previous investigations have also demonstrated that intraoperative strategies aimed at improving CO₂ clearance—such as controlled hyperventilation or pulmonary recruitment maneuvers—may reduce postoperative shoulder pain [12]. Similarly, subdiaphragmatic local anesthetic instillation has been shown to alleviate shoulder-tip pain associated with CO₂ pneumoperitoneum [13]. The observed associations in the present study are consistent with this body of literature, supporting the concept that improved evacuation of intraperitoneal CO₂ may be associated with reduced postoperative discomfort.

However, not all studies have reported consistent benefits, which may be attributable to differences in study design, sample size, patient selection, surgical technique, and postoperative analgesic protocols [14]. Although analgesic management was standardized in the present cohort, several clinically relevant perioperative variables—such as operative difficulty, pneumoperitoneum duration, gallbladder perforation, or extent of peritoneal irrigation—were not systematically recorded. These factors may contribute to interstudy variability and limit direct comparison across studies.

Overall, the existing literature suggests that techniques facilitating more efficient CO₂ evacuation may be associated with improved postoperative comfort; however, the strength and consistency of this association vary across clinical contexts.

### Possible mechanisms

The potential benefit of active CO₂ aspiration may be related to the removal of residual intraperitoneal gas at the end of surgery, thereby reducing peritoneal tension and diaphragmatic irritation. This may limit phrenic nerve stimulation and the reflex pathways implicated in referred shoulder pain. In addition, reduced CO₂ retention may attenuate peritoneal acidosis and inflammatory responses associated with CO₂ absorption, potentially decreasing visceral discomfort [15]. While these mechanisms are biologically plausible and supported by experimental data, they were not directly assessed in the present study and therefore remain inferential.

### Clinical context and interpretation

Postoperative pain may delay early mobilization, increase the risk of pulmonary complications, and prolong hospitalization [16]. Although the present findings suggest an association between active CO₂ aspiration and lower postoperative pain scores, these results should be interpreted cautiously. Pain was assessed using a global VAS, and specific pain components—such as shoulder, incisional, or visceral pain—were not evaluated separately. This approach reflects overall patient-reported pain experience but limits construct validity with respect to individual pain mechanisms.

In addition, routine drain placement in this cohort reflects local institutional practice and should not be interpreted as an endorsement of routine drainage, particularly within Enhanced Recovery After Surgery (ERAS)-based LC pathways. Therefore, the applicability of these findings to settings without routine drainage may be limited.

### Limitations

This study has several important methodological limitations that should be acknowledged. First, the non-randomized prospective design is inherently susceptible to selection bias, as group allocation was determined by a predefined weekly institutional protocol rather than individual patient randomization. As a result, residual confounding due to unmeasured baseline and perioperative variables cannot be excluded, and the observed findings should be interpreted strictly as associations rather than causal effects.

Second, although postoperative pain assessments were conducted by healthcare personnel blinded to group allocation, blinding of patients and surgeons was not feasible due to the nature of the intervention. Therefore, the potential for performance bias and expectancy effects cannot be completely ruled out.

Third, postoperative pain was evaluated using a single global VAS. While this approach reflects routine clinical practice and overall patient-reported pain experience, it does not allow for differentiation between specific pain components such as shoulder, incisional, or visceral pain. Consequently, construct validity with respect to individual pain mechanisms is limited. In addition, objective inflammatory or biochemical markers related to CO₂ absorption or peritoneal irritation were not measured, restricting physiological interpretation of the findings.

Fourth, several clinically relevant covariates—including body mass index (BMI), ASA classification, operative duration, pneumoperitoneum time, severity of gallbladder inflammation, intraoperative perforation, and extent of peritoneal irrigation—were not consistently recorded and therefore could not be incorporated into adjusted analytical models. Although postoperative analgesia was standardized, variability in intraoperative anesthetic and ventilation strategies may have influenced postoperative pain perception. Operative duration was not systematically recorded in a standardized format and therefore could not be analyzed. Operative duration and pneumoperitoneum time may influence postoperative pain and could not be evaluated as potential confounders in this cohort.

Finally, patients with acute cholecystitis and intraoperative gallbladder perforation were deliberately excluded to minimize baseline heterogeneity in postoperative pain outcomes related to inflammatory or chemical peritoneal irritation. While this exclusion improved cohort homogeneity and facilitated a clearer evaluation of pneumoperitoneum-related pain, it may limit the generalizability of the findings to more complex or inflammatory clinical scenarios. An exploratory path analysis is provided in the Supplementary Material and should be interpreted as hypothesis-generating only.

These limitations should be considered when interpreting the observed associations.

### Future directions

Future research should extend the evaluation of active CO₂ aspiration beyond LC to other minimally invasive procedures, including appendectomy, bariatric surgery, and gynecologic laparoscopy, to assess the generalizability of the observed associations. In particular, large-scale, multicenter randomized controlled trials (RCTs) with standardized intraoperative protocols are required to determine whether active CO₂ aspiration provides independent benefit across different surgical contexts.

Future trials should also explore procedural variables such as aspiration pressure, duration, and timing, as well as their interaction with pneumoperitoneum characteristics and anesthetic management. Detailed assessment of pain subcomponents (e.g., shoulder, incisional, and visceral pain) and incorporation of objective inflammatory or biochemical markers would further clarify the physiological mechanisms underlying postoperative pain modulation.

Although the present study identified an association between active CO₂ evacuation and lower postoperative pain scores, its non-randomized design precludes causal inference, and residual confounding cannot be excluded. Therefore, routine incorporation of active CO₂ aspiration into clinical practice should be guided by high-quality randomized evidence rather than observational data alone.

## Conclusion

Active aspiration of residual CO₂ at the end of LC was associated with lower postoperative pain scores at both early and late postoperative time points in this prospective cohort. These findings indicate an observed association between CO₂ evacuation and postoperative pain outcomes; however, they do not establish causality. Given the non-randomized design and the presence of unmeasured confounding factors, the results should be interpreted cautiously. Further well-designed, adequately powered randomized controlled trials are required to determine whether active CO₂ aspiration provides independent clinical benefit across different perioperative settings.

## Supplementary Information

Supplementary Material 1.

## Data Availability

The datasets generated and/or analyzed during the current study are not publicly available due to institutional data protection regulations. However, de-identified data can be made available from the corresponding author upon reasonable request and with permission from Malatya Turgut Özal University institutional authorities.

## Abbreviations

ASA: American Society of Anesthesiologists
BMI: Body Mass Index
CO₂: Carbon Dioxide
LC: Laparoscopic Cholecystectomy
VAS: Visual Analog Scale
JP drain: Jackson–Pratt drain
