# Supplementary material for: Relieving the pressure: the effect of active carbon dioxide aspiration on postoperative pain after laparoscopic cholecystectomy
Source: BMC Surg. 2026 Feb 4;26:180. doi: 10.1186/s12893-026-03567-y (PMC12964736; doi:10.1186/s12893-026-03567-y)
Supplement: Supplementary file 1 — Supplementary Material 1. [file 12893_2026_3567_MOESM1_ESM.docx]

**Path Analysis**

An exploratory path analysis was conducted to descriptively examine associations between active carbon dioxide aspiration, age, sex, and postoperative pain intensity. This analysis was performed exclusively to provide supplementary descriptive insight and was not intended to replace standard regression analyses or to support causal inference.

At the 24th postoperative hour, an inverse association was observed between active gas aspiration and postoperative pain scores (standardized coefficient β = –0.62). Associations between postoperative pain intensity and age or sex were weak and inconsistent across the evaluated models.

The residual variance suggested that a substantial proportion of the variability in postoperative pain scores remained unexplained. Inter-correlations among independent variables were minimal (|r| < 0.10), suggesting no relevant multicollinearity within the model.

Importantly, several clinically relevant variables—including body mass index, ASA classification, operative duration, pneumoperitoneum time, severity of gallbladder inflammation, intraoperative gallbladder perforation, and anesthetic or analgesic variability—were not available for inclusion. Consequently, residual confounding cannot be excluded. The findings of this path analysis should therefore be interpreted strictly as exploratory and hypothesis-generating, and should not be considered confirmatory, predictive, or causal.


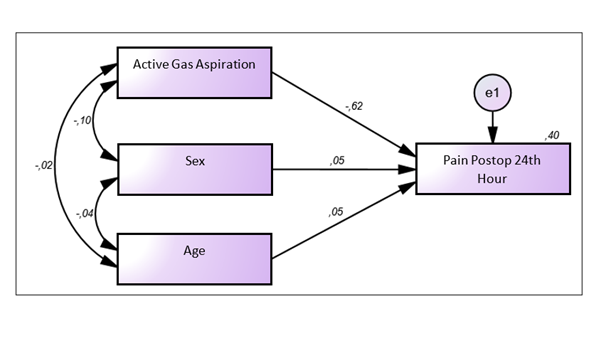


***Supplementary Figure S1*** *Path diagram illustrating exploratory associations between active CO₂ aspiration, age, sex, and postoperative pain intensity.*

*This figure is provided for transparency only and does not imply causality.*

*The residual variance suggested that a substantial proportion of the variability in postoperative pain scores remained unexplained.*

Table 4 summarizes the results of the path analysis evaluating the effects of active gas aspiration, sex, and age on postoperative pain scores.

**Supplementary Table S1. Exploratory path analysis examining associations between aspiration status, age, sex, and postoperative pain scores**

| **Dependent variable** | **Independent variable** | **B** | **β** | **95% CI for B** | **p-value** | **R²** |
| --- | --- | --- | --- | --- | --- | --- |
| 4th-hour pain | Active gas aspiration | -1.76 | -0.54 | –2.06 to –1.45 | <0.001 | 0.332 |
|  | Sex (female) | 0.64 | 0.15 | 0.24 to 1.04 | 0.002 |  |
|  | Age | 0.00 | 0.02 | –0.02 to 0.03 | 0.662 |  |
| 24th-hour pain | Active gas aspiration | -1.88 | -0.62 | –2.21 to –1.55 | <0.001 | 0.398 |
|  | Sex (female) | 0.18 | 0.05 | –0.16 to 0.53 | 0.315 |  |
|  | Age | 0.01 | 0.05 | –0.01 to 0.04 | 0.299 |  |

*Confidence intervals were estimated using bias-corrected bootstrapping with 1000 samples. Results are presented for exploratory purposes only.*
